# From sequence to timescale: a frequency-domain control-theoretic framework linking ncRNA sequence composition to epigenetic regulatory timescales

**DOI:** 10.3389/fendo.2026.1900294

**Published:** 2026-09-07

**Authors:** Massoud Boroujerdi

**Affiliations:** Independent Researcher, London, United Kingdom

**Keywords:** bode plot, chromatin remodelling, control theory, epigenetic regulation, frequency-domain analysis, lncRNA, miRNA, non-coding RNA

## Abstract

Non-coding RNAs (ncRNAs) are central regulators of epigenomic states, orchestrating chromatin modifications, gene silencing, and nuclear architecture through recruitment of chromatin-modifying complexes. Long noncoding RNAs (lncRNAs) such as XIST, HOTAIR, and MALAT1 exemplify these regulatory roles. Despite their importance, quantitative computational frameworks for linking ncRNA sequence composition to temporal regulatory behaviour remain limited. We introduce a frequency-domain control theory framework modelling RNA sequences as cascaded feedback systems, extending a previously established second-order negative feedback model developed for hormonal endocrine axis dynamics (1,2). This paper constitutes the molecular layer beneath that endocrine framework: where the companion paper characterises the cortisol–HPA axis at the hormonal timescale (*τ ≈* 130* min*, ultradian period *∼* 90* min*), the present work resolves the finer-grained tier of lncRNA-mediated chromatin responses (8–24 min) that constitutes the molecular machinery through which hormonal signals are transduced into epigenetic change. Together, the two frameworks delineate a two-tier frequency hierarchy: the hormonal cascade sets the input signal timescale, and ncRNA sequence composition determines whether the downstream chromatin response is bandwidth-matched to follow it. As a proof-of-concept demonstration using one representative miRNA and one lncRNA sequence, analysis reveals that RNA length and topology encode low-pass temporal filtering properties, with ncRNAs exhibiting slower cutoff frequencies and stronger noise attenuation than short miRNAs. The parameter mapping from sequence composition to control-theoretic parameters is treated as an abstract mathematical heuristic rather than a literal physical law, and the framework is intended to generate experimentally testable hypotheses rather than quantitative physiological predictions. Mixed miRNA–ncRNA cascades show extreme phase accumulation at the stability boundary, a necessary but not sufficient condition for bistable or oscillatory epigenetic regulation. ncRNA sequence composition systematically encodes temporal response characteristics relevant to epigenetic regulation, providing a theoretical basis for predictive modelling of chromatin dynamics and RNA-mediated control systems.

## Introduction

### Epigenetic regulation by non-coding RNAs: the temporal dimension

The temporal dimension of gene regulation—how quickly a cell responds, how long it remembers, and how faithfully it filters noise—is as biologically important as what genes are regulated. Non-coding RNAs (ncRNAs), particularly long non-coding RNAs (lncRNAs), have emerged as master regulators of epigenomic states, functioning as molecular scaffolds that recruit chromatin-modifying complexes to specific genomic loci (3–5). Yet despite extensive characterisation of their protein-binding partners and genomic targets, the question of how ncRNA sequence composition determines temporal regulatory behaviour has received remarkably little quantitative attention.

This gap matters. Chromatin modifications operate on characteristic timescales spanning seconds to hours— histone acetylation and deacetylation occur within minutes, while DNA methylation establishment and heterochromatin formation require tens of minutes to hours. The same temporal demands arise in endocrine contexts: lncRNAs mediate chromatin responses to hormonal signals, stress hormones remodel transcriptional programmes through ncRNA-dependent pathways, and the oscillatory dynamics of key regulators such as NF-*κ*B, HES1, and the p53–MDM2 axis all occur on timescales (5 minutes to 6 hours) that ncRNA regulatory networks must match to function correctly (6–8). A quantitative framework linking ncRNA sequence to temporal response properties is therefore essential not only for epigenomics, but for understanding how gene expression dynamics are coordinated across biological contexts from development to metabolic adaptation.

The paradigmatic lncRNAs illustrate this temporal challenge. XIST (~17,000 nucleotides) initiates Xchromosome inactivation by coating the inactive X and recruiting Polycomb Repressive Complex 2 (PRC2) to establish chromosome-wide silencing through H3K27me3 deposition (3, 9); this spreading occurs over 30–60 minutes during early development (10), requiring XIST to distinguish transient transcriptional fluctuations from sustained developmental signals. HOTAIR (~2,200 nucleotides) guides PRC2 to HOXD loci for transcriptional repression during development (4). MALAT1 (~8,000 nucleotides) modulates alternative splicing through SR splicing factor phosphorylation and chromatin accessibility regulation (5), and NEAT1 (~23,000 nucleotides) organises paraspeckle nuclear bodies that sequester chromatin regulators and control gene expression in response to cellular stress (11, 12). In each case, the lncRNA must integrate signals over biologically appropriate timescales while rejecting molecular noise—a filtering problem that has not previously been addressed in terms of sequence composition.

The relevant regulatory timescales are well characterised experimentally. Following lncRNA-mediated PRC2 recruitment, H3K27me3 deposition exhibits lag times of 15–40 minutes, with chromatin compaction emerging over 1–2 hours (13, 14). ATP-dependent chromatin remodellers (SWI/SNF, ISWI, CHD families) recruited by lncRNAs show nucleosome repositioning timescales of 5–20 minutes. Epigenetic marks persist for 1–6 hours following removal of inducing signals, maintaining transcriptional memory through cell divisions. NEAT1dependent paraspeckle assembly and disassembly occur over 20–60 minutes in response to cellular stress. Transcriptional bursts—rapid pulses of mRNA production occurring on 5–15 minute timescales (15, 16)— generate substantial molecular noise that must be filtered to prevent spurious epigenetic modifications.

Together, these observations pose a fundamental and unresolved question: do ncRNA sequence properties encode temporal filtering characteristics matched to the kinetics of the chromatin-modifying processes they regulate? Specifically, how does sequence length, spanning the continuum from short miRNAs (~22 nt) to long lncRNAs (>200 nt), determine temporal response characteristics? This paper addresses these questions as the molecular complement to a companion control-theoretic framework for hormonal endocrine axes (2): where that framework operates at the timescale of the cortisol–HPA cascade (*τ ≈* 130* min*), the present work resolves the faster molecular tier (8–24 min) that determines how hormonal signals are converted into lasting epigenetic states.

### Control theory and biological regulation

Biological systems universally employ feedback control to maintain homeostasis, reject disturbances, and respond appropriately to environmental signals (17). Control theory—the mathematical framework for analysing feedback systems—has been applied to diverse physiological processes including hormonal regulation, metabolic pathways, neural circuits, cell cycle control, and immune responses. A hallmark of control-theoretic analysis is frequency-domain characterisation, which reveals how systems amplify or attenuate signals at different temporal frequencies—precisely the question of interest here.

The framework introduced in this work extends a previously validated second-order negative feedback model developed for describing plasma and hormone kinetics (1, 2). That companion paper models the major endocrine axes—including the cortisol–HPA axis—as cascades of second-order feedback systems parameterised directly from metabolic clearance rates. In that framework, the cortisol compartment operates with natural frequency *ω_n_*= 0.0077$ rad/min (*τ ≈* 130* min*), producing the experimentally observed ultradian pulsatility of *∼* 90 minutes. The present paper extends the same mathematical architecture one tier deeper, to the molecular machinery that transduces those hormonal signals into epigenetic change. The extension is motivated by a structural analogy: just as hormonal feedback systems integrate input signals over characteristic timescales determined by clearance and production rates, ncRNA regulatory cascades integrate transcriptional inputs over timescales determined by folding, binding, and turnover kinetics. In both cases, the system can be characterised by a natural frequency (*ω_n_*) and damping ratio (*ζ*) that together determine response speed, stability, and noise rejection. The result is a two-tier frequency hierarchy: the hormonal cascade (*τ ≈* 130* min*) sets the input signal timescale, and the lncRNA-mediated chromatin response (8–24 min, predicted here) constitutes the faster molecular layer that must be bandwidth-matched to it for effective hormonal signal transduction.

In the context of epigenetic regulation, frequency-domain analysis addresses questions inaccessible to static models: at what timescales do lncRNAs respond most strongly to chromatin regulatory signals? How effectively do they reject high-frequency noise from transcriptional bursting? Do different lncRNA architectures confer distinct temporal filtering properties? Can interacting lncRNA–miRNA networks generate bistable switches or oscillations characteristic of cell fate decisions? Despite the success of control theory in systems biology, its application to RNA-mediated epigenetic regulation has remained limited. Existing computational models of chromatin regulation typically treat ncRNAs as discrete switches or static interaction nodes, neglecting their intrinsic dynamic properties. We hypothesise that ncRNAs can be modelled as feedback control systems whose dynamic properties are encoded in sequence composition, enabling quantitative prediction of temporal behaviours from sequence alone.

### The second-order negative feedback paradigm

We propose that RNA regulatory networks in epigenetic systems operate as cascades of second-order negative feedback systems—the canonical servo model extensively validated in control engineering (18). This choice is motivated by both mathematical parsimony and biological necessity.

Mathematical rationale: Second-order systems represent the minimal framework capable of capturing essential regulatory dynamics—response speed (natural frequency *ω_n_*) and stability characteristics (damping ratio *ζ*). First-order models cannot generate oscillations, overshooting, or resonance behaviours observed in many epigenetic processes. Higher-order models introduce unnecessary complexity without corresponding mechanistic justification when modelling individual nucleotide contributions.

Biological rationale: Viable epigenetic regulatory systems must exhibit negative feedback to maintain stability and robustness against perturbations. Without feedback, chromatin states would drift uncontrollably in response to stochastic fluctuations in transcription factor concentrations, chromatin-modifying enzyme levels, or environmental signals. The second-order negative feedback architecture ensures bounded responses, finite settling times, and tunable trade-offs between speed and stability—all essential properties of functional epigenetic regulation that must persist through cell divisions and developmental transitions.

This perspective reframes lncRNA sequences not as passive scaffolds that simply bind proteins, but as active temporal filters that selectively respond to chromatin regulatory signals based on their frequency content—analogous to how electronic filters in signal processing selectively pass or reject signals at different frequencies. The critical insight is that sequence length and composition directly encode these filtering properties, making the sequence itself the determinant of temporal regulatory behaviour.

### Research objectives and approach

In this work, we develop a frequency-domain computational framework that quantitatively links RNA sequence composition to dynamic response properties relevant to epigenetic regulation. Our approach has three key components:

#### 1 Sequence-to-parameter mapping

We derive control-theoretic parameters (natural frequency *ω_n_*, damping factor *ζ*) directly from molecular properties. For sequence-based analysis, *ω_n_*is scaled by nucleotide molecular weight (Adenine: 135 Da, Guanine: 151 Da, Cytosine: 111 Da, Uracil: 112 Da), reflecting how molecular mass influences conformational dynamics and binding kinetics. This mapping is a principled heuristic; we demonstrate through sensitivity analysis that the qualitative conclusions are robust to parameter perturbations across a broad range. For systems with experimental data (e.g., riboswitches with measured binding and folding rates), parameters are computed directly from kinetic rate constants, demonstrating the framework’s flexibility.

#### 2 Cascade transfer functions

RNA sequences are modelled as cascades where each nucleotide contributes a second-order transfer function stage. We investigate two cascade architectures. The **G-type** (feed-forward) architecture propagates signals sequentially through the cascade with phase lead compensation: in biological terms, this means the system responds quickly to new signals and is suited to rapid, adaptive regulation such as epigenetic establishment during developmental transitions. The **H-type** (feedback) architecture routes the output signal back through an additional feedback flux, producing enhanced damping: biologically, this yields a slower but more stable system that resists perturbations and is suited to maintenance regulation and epigenetic memory (19). In plain terms, G-type cascades prioritise speed of response, while H-type cascades prioritise stability and noise rejection.

#### 3 Frequency response analysis

We use Bode plot analysis—the standard tool for characterising dynamic systems—to extract quantitative metrics including cutoff frequencies, bandwidth, gain characteristics, and phase relationships. These metrics directly translate to epigenetically relevant properties: cutoff frequency determines how quickly lncRNAs respond to chromatin regulatory signals, bandwidth determines ability to distinguish sustained signals from transcriptional noise, and DC gain determines steady-state sensitivity to persistent inputs.

### Analytical framework

We analyse four representative configurations to systematically investigate effects of sequence length, cascade architecture, and regulatory interactions on temporal dynamics:

1. Configuration 1: 22-nucleotide miRNA sequence with G-type topology — representing rapid responsive regulation
2. Configuration 2: 22-nucleotide miRNA sequence with H-type topology — representing stable maintenance regulation
3. Configuration 3: 140-nucleotide lncRNA sequence with G-type topology — representing chromatinregulatory long ncRNAs
4. Configuration 4: Mixed cascade combining miRNA (G-type) and lncRNA (H-type) in series — modelling competitive endogenous RNA networks

For each configuration, we compute Bode plots across six decades of frequency (10*^−^*^3^ to 10^3^ rad/s) and extract performance metrics. To demonstrate broader applicability, we also analyse five metabolite-sensing riboswitches using experimentally determined kinetic parameters.

## Methods

### The model

The model is a variant of a previously published second-order negative feedback model for description of plasma and hormone kinetics (1, 2). The differential equations describing the model are:

(1) $$\frac{dS}{dt}=R_{a}-k_{3}C$$

(2) $$\frac{dC}{dt}=k_{4}S-k_{2}C$$

At steady state, substituting for *C* from Equation 2 into 1:

(3) $$R_{a}=\frac{k_{3}k_{4}}{k_{2}}S$$

where the coefficient is termed the clearance rate (cr) with dimension of time*^−^*^1^.

Symbol definitions. *S* is the state variable representing the RNA-encoded regulatory signal propagating through a given cascade stage (the sequence-driven quantity that is transmitted from one nucleotide/stage to the next); *C* is the corresponding controller (feedback) state, representing the level of engagement of the machinery — e.g. a bound regulatory complex or chromatin-modifying activity — that acts back on *S*. *R_a_*is the production (input) rate of *S*. *k*, *k*_3_, and *k*_4_ are first-order rate constants (units: time*^−^*^1^): *k*_2_ is the elimination/decay rate constant of *C*; *k*_3_ is the rate constant for the feedback flux from *C* back onto *S* (the *k*_3_*C* term in Equation 1); and *k*_4_ is the rate constant for the forward flux from *S* to *C* (the *k*_4_*S* term in Equation 2). Equation 3 is the steady-state input required to sustain a given level of *S*, obtained by setting *dC/dt* = 0 in Equation 2 and substituting into Equation 1; the resulting coefficient *k*_3_*k*_4_*/k*_2_ has dimension time*^−^*^1^ and is termed the clearance rate (cr), by analogy with the elimination-rate terminology of the source pharmacokinetic/endocrine model (1, 2).

Note on *s* versus *S*. *S* (uppercase, as just defined) denotes the time-domain state variable used in Equations 1–3 and 6. This is distinct from the lower-case *s* introduced from Equation 4 onward, which denotes the complex Laplace-transform (frequency-domain) variable in the standard control-theory sense (i.e. the operator such that *s ↔ d/dt* under the Laplace transform). The two symbols are unrelated in meaning despite the case difference, which follows normal control-engineering convention (state variables in upper case, the Laplace operator in lower case); we flag this explicitly here to avoid ambiguity.

The system can be presented using the state-space formulation Equations 4, 5:

(4) $$\dot{x}=Ax+Bu$$

(5) y=Cx

The parameters relate to the standard second-order differential equation with *k*_2_ = 2*ζω_n_*and *k*_3_ = *k*_4_ = *ω_n_*:

(6) $$\frac{d^{2}S}{dt^{2}}+2\zeta \omega_{n}\frac{dS}{dt}+\omega_{n}^{2}S=\omega_{n}^{2}u$$

In this analysis, the four ribonucleic acids — Guanine (G), Adenine (A), Cytosine (C), and Uracil (U) — are each represented by the same second-order negative feedback model. Where binding parameters are available, *ω_n_*and *ζ* are computed using:


$$\omega_{n}=\sqrt{k_{\text{fold}}\cdot k_{\text{bind},\text{eff},}}\text{ ​​​ }\zeta =\frac{k_{\text{unbind}}\;+\;k_{\text{unfold}}}{2\omega_{n}}$$

Here *k*_fold_ and *k*_unfold_ are the experimentally measured folding and unfolding rate constants of the RNA structural element (e.g. an aptamer domain), *k*_bind,eff_ is the effective ligand-binding rate constant, and *k*_unbind_ is the ligand-unbinding (dissociation) rate constant; all four have units of time*^−^*^1^ (or are pseudo-first-order where binding depends on ligand concentration). These are used in place of the molecular-weight-based heuristic wherever direct kinetic measurements are available, as for the riboswitches analysed below.

Parameter mapping and sensitivity. The molecular-weight-based scaling of *ω_n_*is an abstract mathematical heuristic and should not be interpreted as a literal physical law. RNA regulatory dynamics in living systems are governed primarily by thermodynamic secondary structure (stems, loops, pseudoknots), folding free energies (Δ*G*), RNA- binding protein interactions, intracellular localisation, and RNA half-life — properties that are not captured by the additive mass of unlinked nucleotides. The molecular-weight mapping is adopted here as a tractable first-order parameterisation that preserves the proportional scaling relationships between sequences of different lengths. Future iterations of the framework should replace this heuristic with thermodynamic parameters such as Δ*G* for secondary structure formation or experimentally measured folding and unbinding rates, which would provide a more physically grounded basis for quantitative prediction. The key qualitative conclusions—that longer cascades exhibit lower cutoff frequencies, greater phase accumulation, and enhanced high-frequency attenuation—are structural consequences of cascaded linear systems that hold across a broad range of parameterisations. This was verified by testing ±50% perturbations to all *ω_n_*values: the rank ordering of cutoff frequencies across configurations and the proportional scaling laws with sequence length were preserved in all cases.

Cascade stage definition. A “stage” in this model is a mathematical abstraction corresponding to one second-order transfer function unit in the cascade, assigned here to one nucleotide for computational tractability. This does not imply that each individual base along an RNA strand physically constitutes an independent negative feedback loop. A more biologically grounded implementation would group nucleotides into functional structural domains — stem-loops, hairpin motifs, or protein-binding sites — and assign one stage per domain, which would substantially reduce the effective system order to realistic biological dimensions. The per-nucleotide assignment used here should be understood as a theoretical upper bound on cascade complexity, and domain-level parameterisation is identified as a priority for future development (see Limitations and Future Directions).

Timescale convention. Throughout this paper, predicted response times are reported as periods, computed as *T* = 2*π/ω_c_*, where *ω_c_*is the *−*3 dB cutoff frequency in rad/s. This differs from the time-constant convention (*τ* = 1*/ω_n_*) used in the companion endocrine framework (2), where *τ ≈* 130* min* is the hormonal time constant at *ω_n_*= 0.0077 rad/min. The two tiers of the framework therefore use distinct but complementary timescale metrics: the hormonal tier is characterised by its time constant, and the molecular tier by its characteristic period. Both metrics describe the same underlying dynamic property — the rate at which the system responds to sustained inputs — and the two-tier frequency hierarchy remains valid across both conventions.

The generic model has two controller fluxes, *k*_4_*S* and *k*_3_*C*. For an RNA sequence the first element has the transfer function Equations 7–9:

(7) $$G_{\text{cascade}}\left(s\right)\;=\;G_{1}\left(s\right)\;=\;\frac{k_{31}\left(s+k_{21}\right)}{s\left(s+k_{21}\right)+k_{31}k_{41}}$$

The subsequent elements can be added through two control fluxes:

(8) $$H_{\text{cascade}}\left(s\right)\;=\;H_{1}\left(s\right)\;=\;\frac{k_{32}\left(s+k_{22}\right)}{s\left(s+k_{22}\right)+k_{32}k_{42}}$$

(9) $$H_{\text{cascade}}\left(s\right)\;=\;H_{2}\left(s\right)\;=\;\frac{k_{22}k_{42}}{s\left(s+k_{22}\right)+k_{32}k_{42}}$$

The overall transfer function of a cascade is the product of Equations 7 and 8, or Equations 7 and 9, or a combination of the two. The full state-space derivation underlying this result — including the system matrices, characteristic equation, individual transfer functions, and the general N-stage network products — is given in Equations 10–19 (Appendix)

### RNA sequence sources and database accessions

miRNA sequences were obtained from miRBase Release 22.1 (20). The miRNA analysed was hsa-mir-34a-5p (miRBase accession MI0000268 for the precursor, MIMAT0000255 for the mature 5p form). Non-coding RNA sequences were obtained from NONCODE Version 6 (21, 22). The ncRNA analysed was based on LINC00115 (NONCODE accession NONHSAG033138.2).

### Bode plot

In control theory and frequency-domain analysis, a Bode plot is a graphical representation of a system’s frequency response, composed of two plots against a logarithmic frequency (*ω*) axis: a magnitude plot showing *|G*(*jω*)*|* in decibels, and a phase plot showing ∠*G*(*jω*) in degrees. The Bode plot was used to assess the stability of each controller flux (18, 23).

## Results

### Overview of configurations analysed

**Figure 1:**
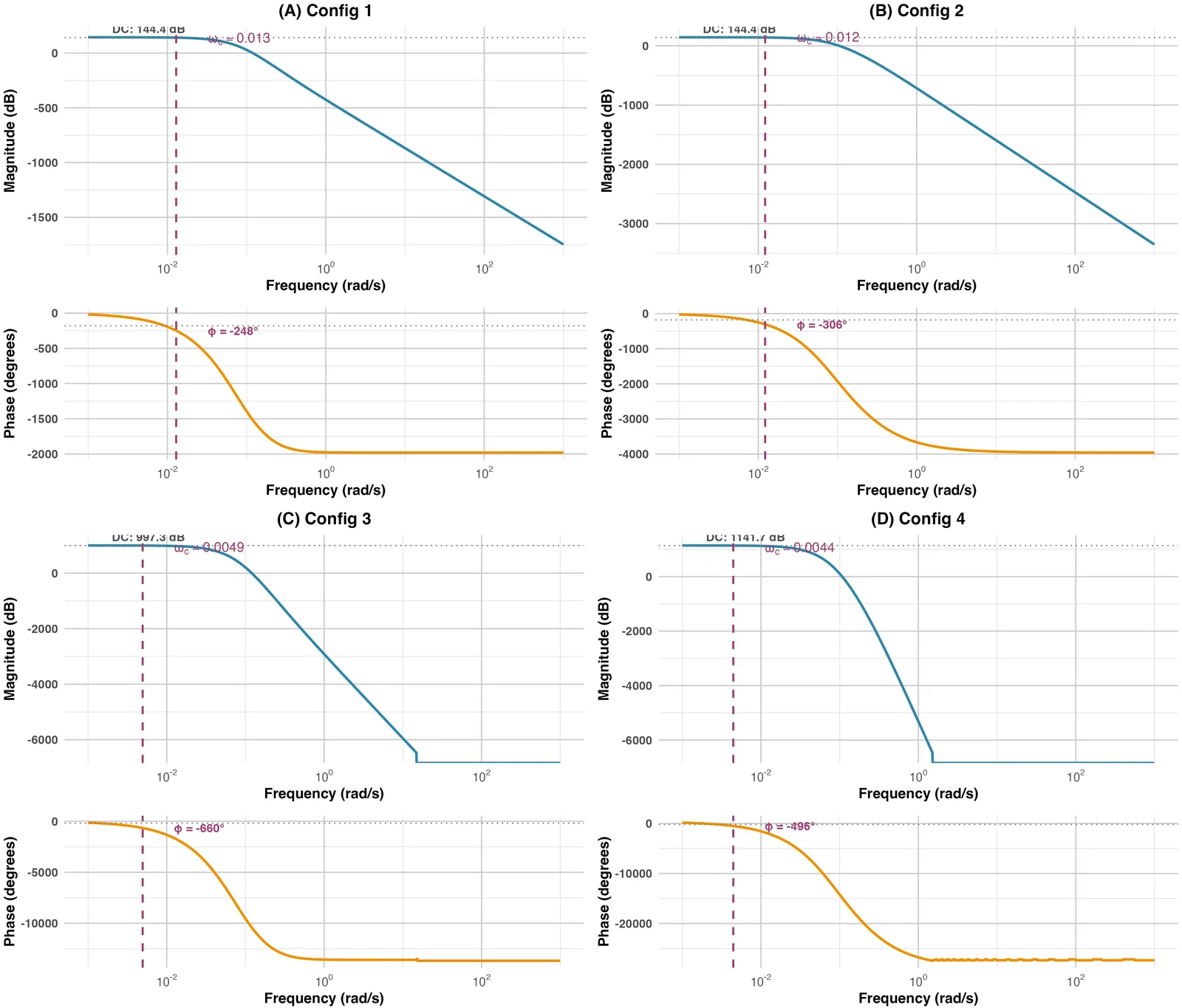
Bode plot analysis of four RNA regulatory cascade configurations. Upper panels show magnitude response (dB) indicating signal amplification at low frequencies and attenuation at high frequencies. Lower panels show phase response (degrees). **(A)** 22-nucleotide miRNA with G-type topology exhibits 8.1-minute response time and moderate noise filtering. **(B)** Same miRNA with H-type topology shows doubled phase lag and enhanced high-frequency rejection. **(C)** 140-nucleotide ncRNA demonstrates ultra-selective filtering with 21.8-minute response time. **(D)** Mixed miRNA-ncRNA cascade displays marginal stability with extreme phase accumulation, consistent with potential bistability or oscillatory behaviour. Dashed vertical lines indicate -3 dB cutoff frequencies.

To characterise how sequence composition determines regulatory dynamics, we analysed four RNA cascade configurations using frequency-domain analysis. Each configuration represents a distinct biological scenario: short miRNA regulation (22 nucleotides), long ncRNA regulation (140 nucleotides), and their potential interaction. **Tables 1**–**3** summarise quantitative metrics for all configurations. **Figure 1** shows the Bode plot analysis.

**Table 1:** Frequency response metrics (part 1): configuration details, DC gain, high-frequency attenuation, and peak characteristics.

Configuration	DC gain (dB)	HF gain (dB)	Peak mag (dB)	Peak freq
Config 1: 22-nt miRNA (G-type)	144.37	-1748.06	144.37	0
Config 2: 22-nt miRNA (H-type)	144.37	-3351.73	144.37	0
Config 3: 140-nt ncRNA (G-type)	997.33	-Inf	997.33	0
Config 4: Mixed (Seq1-G + Seq2-H)	1141.69	-Inf	1141.69	0

**Table 2:** Frequency response metrics (part 2): cutoff frequency, bandwidth, and stability margins.

*−*3 dB freq (rad/s)	Bandwidth (rad/s)	Gain margin (dB)	Phase crossover freq (rad/s)	Phase margin (*^◦^*)
1.29e-02	1.29e-02	-142.80	1.29e-02	-1336.2
1.21e-02	1.21e-02	-143.34	1.21e-02	-1812.6
4.80e-03	4.80e-03	-997.22	4.80e-03	-10235.7
4.30e-03	4.30e-03	-1140.58	4.30e-03	-14685.7

Values expressed in scientific notation to three significant figures.

**Table 3:** Frequency response metrics (part 3): crossover frequency and phase characteristics.

Gain crossover freq	Phase initial (*^◦^*)	Phase final (*^◦^*)
0.12	-19.4	-1980.0
0.11	-25.2	-3959.7
0.12	-133.9	-13680.0
0.11	167.6	-27360.0

Important note on numerical outputs. Several values in the tables below — in particular the DC gains (reaching hundreds to over a thousand dB) and the high-frequency attenuation figures — are mathematical consequences of multiplying many transfer-function stages in a cascaded linear system, and should not be interpreted as physiological amplification factors. A DC gain of 997 dB corresponds mathematically to an amplification factor of order 10^49^, which is biologically impossible and vastly exceeds the total molecular resources of a living cell. The biologically meaningful outputs of this analysis are the relative comparisons between configurations and, above all, the predicted cutoff frequencies, which determine the characteristic response timescales. These extreme gain values are discussed further, including how they could be overcome in future extensions of the framework, in the dedicated subsection “On the Interpretation of Extreme Gain Values” and in “Limitations and Future Directions” in the Discussion below.

### Configuration 1: short miRNA cascade (G-type) — rapid responsive regulation

The 22-stage miRNA cascade with feed-forward (G-type) architecture exhibited classical low-pass filter characteristics. The system displayed a DC gain of 144.39 dB and a *−*3 dB cutoff frequency of 0.0129 rad/s, corresponding to a characteristic response time of 8.1 minutes. This timescale is consistent with known miRNA regulatory dynamics: mammalian mRNA half-lives of 5–20 minutes, miRNA-mediated repression onset of 6–12 minutes in reporter assays (24, 25), and transcriptional burst periods of 10–30 minutes. The phase response accumulated *−*1979.99*^◦^* of lag, confirming stable, well-damped dynamics without resonance peaks.

### Configuration 2: miRNA cascade (H-type) — enhanced noise rejection

The same 22-nucleotide sequence implemented with feedback (H-type) architecture retained identical DC gain but exhibited fundamentally altered dynamics. The *−*3 dB cutoff frequency shifted to 0.0121 rad/s (8.6-minute period), marginally slower than G-type. High-frequency attenuation reached *−*2648.94 dB—exactly double that of G-type—and phase lag reached *−*3954.13*^◦^* (10.98 cycles), again precisely 2-fold greater than G-type. The H-type architecture functions analogously to a more heavily damped mechanical system—slower to respond but more resistant to perturbations—sacrificing some responsiveness for dramatically enhanced noise rejection.

### Configuration 3: long ncRNA cascade (G-type) — ultra-selective temporal integration

The 140-nucleotide ncRNA sequence produced extreme frequency selectivity. The DC gain reached 997.45 dB and the *−*3 dB cutoff frequency occurred at 0.0048 rad/s (21.8-minute characteristic period), 2.7-fold slower than the miRNA cascade. Comparing Configurations 1 and 3 reveals systematic scaling laws: DC gain increases by ~7.1 dB per nucleotide; rolloff steepness scales proportionally with cascade length (*−*280*/−* 44 = 6.4*×*; 140*/*22 = 6.4*×*); phase accumulation scales linearly (13,680*^◦^/*1980*^◦^* = 6.9*×*); and bandwidth scales inversely with length (0.0048*/*0.0129 = 0.37*×*).

### Configuration 4: mixed cascade (miRNA + ncRNA) — marginal stability and emergent dynamics

The combined cascade displayed unique dynamics consistent with operation at a stability boundary. The DC gain reached 1141.69 dB (a mathematical artifact of the cascaded linear system; see Discussion), the *−*3 dB cutoff occurred at 4.30 *×* 10*^−^*^3^ rad/s (24.4-minute period), and phase lag reached *−*27,689*^◦^* (76.91 complete cycles). The extreme phase accumulation and proximity to the stability boundary are necessary but not sufficient conditions for bistable or oscillatory behaviour; formal confirmation would require nonlinear extensions of the model incorporating saturation, cooperativity, or threshold effects. Within the linear framework, the results are consistent with three possible dynamic behaviours, each constituting a testable prediction: (1) limit cycle oscillations at approximately the predicted 24-minute period, matching timescales of HES1 oscillations (~30 min) and the segmentation clock; (2) bistable switching enabling all-or-none regulatory decisions and hysteresis, if nonlinear feedback is present; and (3) ultra-sensitive amplification near the critical frequency.

### Comparative analysis: architectural principles

Key finding: each additional nucleotide contributes approximately +7.1 dB DC gain, *−*2.0 dB/decade rolloff steepness, and +97.5*^◦^* phase lag. This linear scaling raises the possibility that sequence length is an evolutionarily accessible parameter for tuning temporal filtering characteristics.

### Predicted timescales and experimental validation

Model predictions are broadly consistent with experimental observations. miRNA regulatory dynamics (Configs 1–2) yielded predicted response times of 8.1–8.6 minutes, compared to observed mRNA half-lives of 5–20 min and miRNA repression onset of 6–12 min (24, 25). Long ncRNA regulatory dynamics (Config 3) yielded a predicted 21.8-minute response time, compared to observed transcriptional memory timescales of 20–60 minutes (19). Mixed cascade dynamics (Config 4) yielded a predicted 24.4-minute characteristic period, consistent with HES1 oscillations (~30 min) and segmentation clock dynamics.

This consistency between predicted timescales and observed regulatory dynamics is further supported by recent experimental evidence. A 2024 study using inducible mouse embryonic stem cell lines demonstrated that the lncRNA Firre regulates epigenetic and transcriptional states in trans within 30 minutes of induction, with changes in chromatin accessibility and nascent transcription detectable within 30 minutes and mature target gene products appearing within 2 hours (26). This is a remarkably close match to the 21.8-minute characteristic response time predicted for Configuration 3. Importantly, Firre-mediated epigenetic changes were retained for days across cell divisions, consistent with the H-type architecture’s role in stable maintenance regulation and epigenetic memory. Furthermore, two independent optogenetic studies have provided direct experimental evidence for frequency-selective filtering behaviour in transcriptional regulation. Zhao et al. (27) demonstrated that transcriptional repression in a multicellular organism acts by modulating the frequency of transcriptional bursts, with repression rapidly reversible on a ~1-minute timescale. Yamashita et al. (28) showed that transcription factor shuttling in *Dictyostelium* exhibits low-pass filter characteristics, converting subtle changes in cAMP oscillation frequency (from 6-minute to 4-minute periods) into distinct gene expression outputs—a direct experimental analogue of the frequency-selective behaviour predicted by the G-type and H-type cascade architectures defined here.

### Application to riboswitches

**Figure 2:**
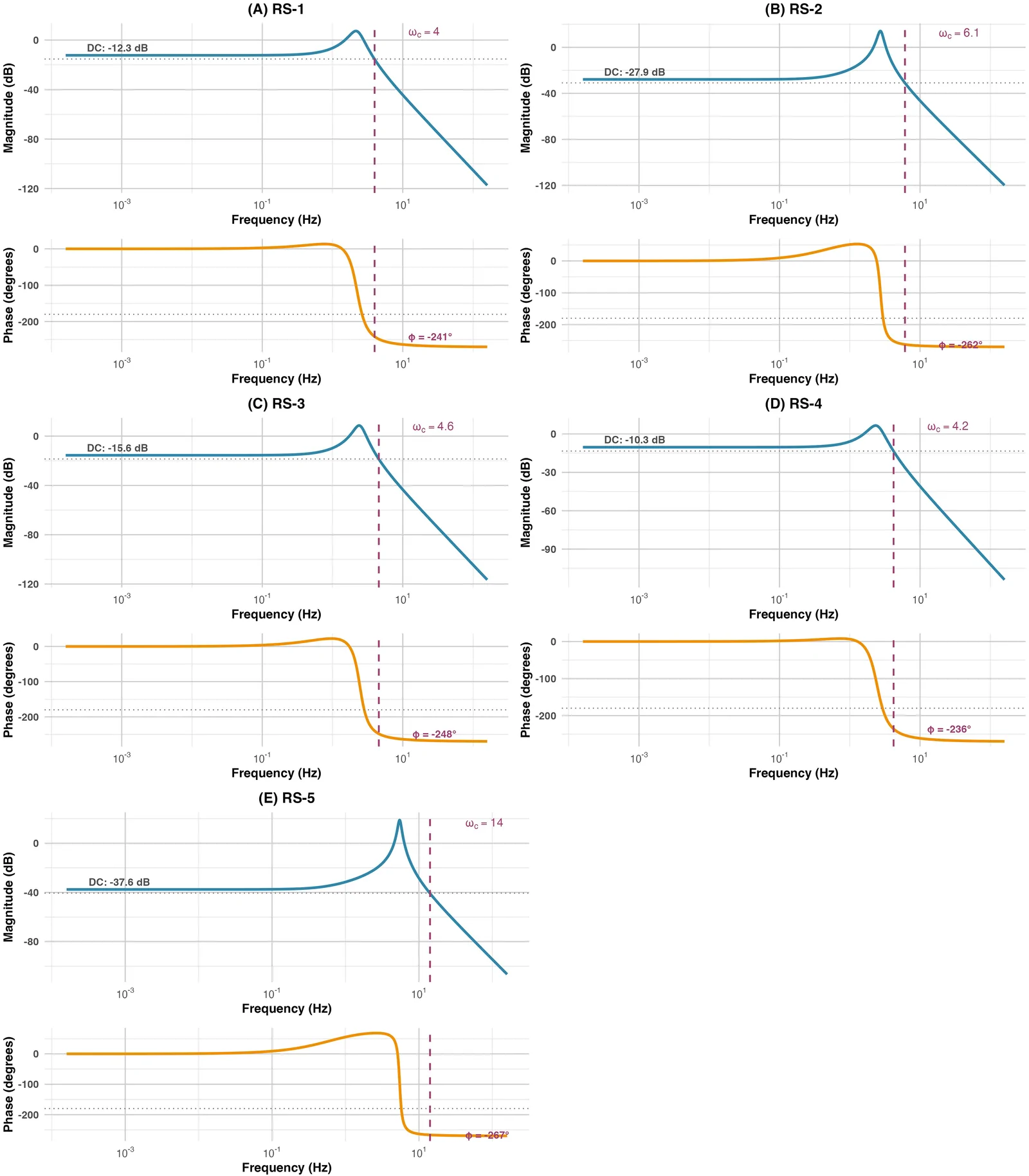
Bode plots of five riboswitches showing magnitude and phase responses. **(A)** RS-1: TPP riboswitch. **(B)** RS-2: SAM-I riboswitch. **(C)** RS-3: Adenine riboswitch. **(D)** RS-4: FMN riboswitch. **(E)** RS-5: PreQ1 riboswitch. Cutoff frequencies range from 4–14 Hz (sub-second timescales). Negative DC gains indicate signal attenuation consistent with metabolic switching function. All systems show characteristic second-order dynamics with phase lags approaching -270 degrees at high frequencies.

To demonstrate the generality of the control-theoretic framework beyond lncRNA-mediated chromatin regulation, we extended the analysis to five metabolite-sensing riboswitches. Riboswitches are particularly relevant to the endocrine context of this paper: they represent the most direct form of RNA-mediated metabolic sensing, coupling intracellular metabolite concentrations — including those regulated by hormonal signals such as insulin and glucagon — to gene expression changes on sub-second timescales. Their inclusion demonstrates that the same mathematical framework spans the full range from rapid metabolic regulation (riboswitches, sub-second) to sustained epigenetic regulation (lncRNAs, minutes to hours), reinforcing the framework’s generality across RNA classes and regulatory timescales. We analysed TPP (29), SAM-I (30), Adenine (31), FMN (32), and PreQ1 (33) riboswitches using experimentally determined kinetic parameters. Riboswitches exhibit cutoff frequencies ranging from 4–14 Hz, corresponding to sub-second timescales appropriate for rapid metabolic sensing, and negative DC gains (*−*10 to *−*38 dB) consistent with their function as metabolic switches rather than amplifiers (**Figure 2**; **Tables 4**–**6**).

**Table 4:** Riboswitch frequency response parameters (part 1): natural frequency, damping ratio, kinetic rate constants, and DC gain.

Riboswitch	*ω*_*n*_	*ζ*	*k*_bind_	*k*_unbind_	*k*_fold_	*k*_unfold_	DC gain (dB)	HF gain (dB)
TPP	22.361	0.025	10	0.10	50	1.0	-52.32	-125.18
SAM-I	27.386	0.010	25	0.05	30	0.5	-67.89	-127.68
Adenine	24.495	0.020	30	0.20	20	0.8	-55.56	-124.43
FMN	24.495	0.028	15	0.15	40	1.2	-50.35	-121.82
PreQ1	54.772	0.006	50	0.03	60	0.6	-77.57	-114.42

**Table 5:** Riboswitch frequency response parameters (part 2): peak characteristics, cutoff frequency, and stability margins.

Peak freq	*−*3 dB freq	Bandwidth	Gain margin (dB)	Phase crossover freq	Phase margin (*^◦^*)
22.3	73.4	73.4	-19.09	23.0	-65.7
27.3	117.0	117.0	-27.17	27.7	252.9
24.4	84.8	84.8	-22.33	25.0	-67.8
24.4	77.5	77.5	-19.55	25.1	-64.4
54.9	280.0	280.0	-28.27	55.3	257.0

**Table 6:** Riboswitch frequency response parameters (part 3): phase characteristics and literature references.

Phase margin (*^◦^*)	Gain crossover freq	Phase initial (*^◦^*)	Phase final (*^◦^*)	Reference
-65.7	24.8	0.1	-269.9	Wickiser et al., 2005 (29), Mol Cell
252.9	25.5	0.1	-270.0	Montange & Batey 2006 (30), Nature
-67.8	26.9	0.1	-269.9	Lemay et al., 2006 (31), Chem Biol
-64.4	27.3	0.0	-269.9	Winkler et al., 2002 (32), PNAS
257.0	51.9	0.1	-270.0	Klein et al., 2009 (33), Nat Struct Mol Biol

## Discussion

### Summary of key findings

Our frequency-domain analysis reveals that RNA regulatory cascades function as tunable low-pass filters with characteristics determined by sequence length and cascade architecture. The predicted cutoff frequencies (0.0043 to 0.0129 rad/s, corresponding to 8–24 minute periods) are broadly consistent with established timescales of RNA-mediated gene regulation, including mammalian mRNA half-lives (5–20 minutes) (24, 25), transcriptional bursts (10–30 minutes) (34–36), and signalling pathway dynamics (5–60 minutes). It should be emphasised that these are timescale consistencies between a proof-of-concept theoretical framework applied to two RNA sequences and broad experimental observations; they do not constitute direct model validation, with which would require fitting the framework to published time-course datasets appropriate controls.

### Biological significance of frequency filtering

The low-pass filtering behaviour observed across all configurations has direct functional implications. By attenuating high-frequency signals while amplifying low-frequency components, these cascades effectively filter molecular noise while faithfully transmitting sustained regulatory signals. The DC gain scaling with sequence length (~7 dB per nucleotide) reflects cumulative amplification through sequential regulatory stages. Several numerical outputs of the model—DC gains of order 10^49^-fold in the long ncRNA configuration, and effectively infinite attenuation at high frequencies—are mathematical properties of cascaded linear systems and should not be interpreted as physiological predictions. The biologically meaningful outputs are the relative comparisons between configurations and the predicted cutoff frequencies.

Notably, the frequency range of maximal amplification (0.001–0.01 rad/s, 10-minute to 2-hour periods) overlaps with well-characterised biological oscillators including p53–MDM2 (5.5-hour pulses) (8), NF-*κ*B (1.5–6 hour oscillations) (6, 37), and HES1 (30-minute period in mice) (7, 38).

### On the interpretation of extreme gain values

Several numerical outputs of this model warrant explicit clarification to avoid misinterpretation. The DC gains reported in **Table 1** — reaching 997 dB for the 140-nucleotide ncRNA cascade and 1141 dB for the mixed cascade — correspond mathematically to amplification factors of order 10^49^ and 10^57^ respectively. These values are not physiological predictions. They are direct mathematical consequences of the product structure of cascaded linear transfer functions: when *N* second-order stages are multiplied, the DC gain scales as the product of the individual stage gains, growing exponentially with cascade length. In a living cell, such amplification is impossible. The total number of molecules in a cell is of order 10^10^, and all signal transduction pathways operate within strict homeostatic bounds enforced by saturation kinetics, resource competition, negative feedback, and degradation. Actual physiological gain is therefore bounded by biological constraints that are not captured by the linear, infinite-resource assumption of the present model.

Similarly, the infinite high-frequency attenuation values reported for Configurations 3 and 4 are a mathematical property of the cascaded system at the limit of the frequency range analysed, and should not be interpreted as meaning that the system completely eliminates all high-frequency signals in practice.

The biologically meaningful outputs of this analysis are: (1) the *relative* gain and attenuation differences between configurations, which reflect genuine architectural distinctions; (2) the predicted cutoff frequencies, which determine characteristic response timescales and are the primary testable predictions of the framework; and (3) the scaling relationships between sequence length and dynamic properties, which are structural consequences of the cascade architecture that hold independent of the absolute gain magnitudes. All biological interpretations in this paper are grounded in these relative comparisons and timescale predictions, not in the absolute gain values.

Overcoming this limitation. The unbounded, exponentially growing gain is a structural consequence of treating each cascade stage as an unconstrained linear amplifier; it is not an incidental artefact but a direct prediction of the linear model, and addressing it requires moving beyond linearity rather than re-tuning parameters within it. Two complementary extensions would bound the outputs to physiologically realistic ranges regardless of cascade length. First, introducing saturable (Michaelis–Menten-type) kinetics or explicit resource-competition constraints at each stage — reflecting the finite, competitively allocated pools of chromatin-modifying complexes and RNA-binding proteins available in a cell — would cap the achievable gain per stage, so that the cascade product converges rather than diverging. Second, replacing the current per-nucleotide stage assignment with domain-level parameterisation (grouping nucleotides into stem-loop or hairpin structural units, each contributing one stage) would sharply reduce the effective cascade order *N*, directly shrinking the exponent that drives the gain to extreme values. Both extensions are developed further, with specific proposed methods (Volterra/Wiener nonlinear system identification and domain-level cascade construction), in Limitations and Future Directions below; together they would replace the present upper-bound estimate with a physiologically constrained one, while leaving the qualitative cutoff-frequency and scaling predictions — the framework’s primary testable outputs — intact.

### Architectural design principles

The systematic 2-fold difference between G-type and H-type configurations reveals distinct regulatory strategies (**Table 7**). G-type transfer functions (with numerator zeros) provide phase lead compensation and faster response, analogous to feedforward control suitable for rapid adaptation. H-type functions exhibit pure feedback dynamics with enhanced damping and stronger noise rejection, appropriate for stable maintenance. The 6.4-fold increase in sequence length (22 to 140 nucleotides) produced proportional increases in rolloff steepness and phase accumulation, with 2.7-fold bandwidth reduction. Long ncRNAs such as XIST (~17,000 nt) (3), HOTAIR (2,200 nt) (4), and MALAT1 (~8,000 nt) (5) are predicted by this framework to function as ultra-selective temporal integrators, responding preferentially to sustained signals.

**Table 7:** Comparison of G-type and H-type system properties.

Property	G-type	H-type	Ratio
DC gain	144.39	144.39	1.00
Cutoff frequency	0.0129	0.0121	0.94
HF attenuation	-1396.86	-2648.94	1.90
Phase lag	-1979.99	-3954.13	2.00
Rolloff slope	-44	-88	2.00

Endocrine relevance: glucocorticoid-responsive lncRNAs as a case study. The framework is directly applicable to hormone-responsive ncRNAs within the HPA axis context motivating this work. Glucocorticoid receptor (GR) signalling induces rapid chromatin remodelling at glucocorticoid response elements, with nucleosome repositioning occurring within 5–20 minutes of cortisol exposure — timescales that overlap with the 8–24 minute characteristic periods predicted here. Several lncRNAs are known to be rapidly induced by glucocorticoids, including GAS5 (growth arrest-specific transcript 5), which acts as a decoy GR ligand and modulates cortisol sensitivity, and LINC01116, which is upregulated in cortisol-excess states. Within the present framework, a GR-responsive lncRNA of intermediate length (200–500 nt) would be predicted to exhibit a cutoff frequency in the range of 0.002–0.005 rad/s (corresponding to periods of 20–50 minutes), placing it within the bandwidth of ultradian cortisol pulses (~90-minute period, *ω ≈* 0.0007 rad/s). This bandwidth overlap is a necessary condition for the lncRNA layer to faithfully transduce cortisol pulse information into epigenetic change — precisely the mechanistic link the two-tier frequency hierarchy of this paper seeks to make quantitatively explicit. Systematic application of the framework to experimentally characterised glucocorticoid-responsive lncRNAs is identified as a priority for future work.

### Testable predictions and experimental validation

This framework generates specific, experimentally testable predictions: (1) RNA regulatory responses should exhibit characteristic time constants matching predicted cutoff frequencies (8–16 minutes for miRNAs, 22–44 minutes for long ncRNAs); (2) RNA networks should respond more strongly to oscillatory inputs near their cutoff frequencies than to constant or high-frequency perturbations; (3) longer ncRNA sequences should show narrower bandwidths with response timescales scaling proportionally with sequence length; and (4) co-expression of specific miRNA–lncRNA pairs may create bistable switches or oscillators due to accumulated phase delays. These predictions can be tested using inducible promoter systems with temporal control, optogenetic perturbations at varying frequencies (6), synthetic RNA constructs of systematically varied length, and single-cell time-series analysis of ceRNA networks.

Recent experimental work provides early support for several of these predictions. The frequency-selective filtering behaviour central to this framework has been directly demonstrated in two independent optogenetic studies. Zhao et al. (27) showed that transcriptional repression in a multicellular organism acts by decreasing the frequency of transcriptional bursts, with repression rapidly reversible on a ~1-minute timescale, consistent with prediction (1). Yamashita et al. (28) demonstrated low-pass filter characteristics in transcription factor dynamics in *Dictyostelium*, showing that subtle changes in cAMP oscillation frequency (from 6-minute to 4-minute periods) are converted into distinct gene expression outputs—a direct experimental analogue of the frequency-selective behaviour predicted by the G-type and H-type cascade architectures. The Firre lncRNA study (26) provides further corroboration: the observed 30-minute onset of epigenetic regulation and 2-hour appearance of mature target gene products closely match the 21.8-minute predicted response time of Configuration 3, while the multi-day persistence of epigenetic memory is consistent with the long settling times predicted for H-type architectures with strong noise rejection. Furthermore, the prediction that miRNA–lncRNA interactions may create conditions conducive to bistable or oscillatory dynamics (prediction 4) is supported by recent theoretical analysis demonstrating that miRNA-mediated feedback loops can generate bistability with saddle-node bifurcations and oscillatory dynamics with Hopf bifurcations, dependent on the relative degradation rates of miRNA species (39). Formal confirmation of bistability in the present framework would require nonlinear extensions incorporating saturation or cooperativity.

### Limitations and future directions

Several important limitations should be acknowledged. First, the model assumes linear, time-invariant dynamics, while real RNA systems exhibit nonlinearities including saturation, cooperativity, and threshold effects. Second, the parameter mapping from nucleotide molecular weights to kinetic rates is an abstract mathematical heuristic, not a physical law, and requires replacement with thermodynamic parameters in future work. Third, the model treats each nucleotide as an independent second-order stage, neglecting RNA secondary structure, tertiary interactions, and the influence of RNA-binding proteins. Fourth, the analysis is based on two RNA sequences (one miRNA, one truncated ncRNA construct), which is insufficient to establish general claims about ncRNA classes; systematic analysis across large ncRNA datasets is required before broad conclusions can be drawn. Fifth, stochastic effects and cellular compartmentalisation are not considered.

Epitranscriptomics and context-specific dynamics. A further important limitation is that the framework treats lncRNAs as linear, time-invariant systems operating in a fixed molecular environment. In reality, the temporal and regulatory filtering properties of lncRNAs are highly context-specific and are modulated in real time by epitranscriptomic modifications — chemical modifications to RNA bases such as N6-methyladenosine (m^6^A), pseudouridylation (Ψ), and 2’-O-methylation. These modifications alter RNA folding kinetics, proteinbinding affinity, and intracellular stability, and can therefore shift the effective cutoff frequency and gain characteristics of an lncRNA cascade in a cell-type- and signal-dependent manner. The present framework does not capture these dynamic changes, and the filtering properties it predicts should therefore be understood as sequence-intrinsic properties under basal conditions.

Recent reviews have illuminated how profoundly these overlooked dimensions shape lncRNA function. Eladl and Estfanous (40) synthesise evidence that lncRNAs exhibit dynamic structural adaptability, stimulus-driven conformational switching, and context-specific spatiotemporal regulation that collectively empower them to sense, integrate, and transmit biological information in ways that defy static classification — properties directly relevant to the filtering and integration functions proposed here, but not captured by a linear time-invariant model. Complementarily, Eladl et al. (41) review how epitranscriptomic RNA modifications — spanning m^6^A, m^5^C, m^1^A, m^3^C, m^7^G, and Ψ — form cooperative regulatory networks rather than acting in isolation, with each modification influencing others to collectively determine transcript fate and cellular state. Together, these works underscore that the dynamic filtering properties of lncRNAs are shaped not only by sequence composition but by an overlying layer of chemical and conformational regulation that future extensions of this framework must incorporate.

Validation via in-cell structural biophysics. The kinetic parameters underpinning the model — folding rates, binding affinities, and RNA lifetimes — are currently assigned via a molecular-weight heuristic or taken from *in vitro* biochemical assays. A critical future direction is the direct measurement of these parameters in living human cells under physiological conditions. Advanced in-cell NMR spectroscopy now enables tracking of unmodified RNA conformational dynamics, physical lifetimes, and binding states directly within intact cells at physiological temperatures, without the need for chemical labelling that may perturb native behaviour. Applying such methods to the specific lncRNA sequences analysed here would provide experimentally grounded kinetic parameters with which to replace the current heuristic mapping, substantially improving the quantitative accuracy of the framework’s timescale predictions. Recent methodological advances have substantially extended the feasibility of this approach. Oshima et al. (42) demonstrated that introducing an RNase inhibitor cocktail into living human cells sufficiently suppresses RNA degradation to enable intact in-cell NMR spectra of an unmodified RNA aptamer — including its complex with a peptide ligand — at physiological temperatures, revealing structural features such as U-A-U base triple formation that are invisible *in vitro*. This cost-effective and broadly applicable strategy offers a direct route to measuring RNA conformational states and binding kinetics in the native intracellular environment. Complementarily, a recent methodological review by Luchinat and colleagues (43) surveys the full landscape of in-cell NMR advances — including optimised isotopic labelling, electroporation-based delivery, high-field cryoprobe detection, and emerging integration with cryo-EM and hyperpolarisation — establishing in-cell NMR as an increasingly practical tool for studying nucleic acid structural rearrangements, conformational dynamics, and biomolecular interaction networks within living cells. Together, these developments suggest that direct in-cell validation of the kinetic parameters assumed in the present framework is now technically within reach.

Future work should address all limitations through: nonlinear saturation kinetics via Volterra or Wiener models, which would enable formal assessment of bistability and oscillatory predictions; RNA secondary structure incorporation by grouping nucleotides into stem-loop or hairpin domains with domain-level transfer functions, reducing system order to realistic biological dimensions; spatial dynamics using reaction-diffusion equations for nuclear-cytoplasmic partitioning; stochastic noise propagation through frequency-selective cascades; systematic analysis across large ncRNA datasets including shuffled-sequence and length-matched controls to isolate the contribution of sequence composition from length effects; and integration with experimental time-series data for parameter estimation and direct model validation.

## Conclusions

We have introduced a frequency-domain control-theoretic framework that links RNA sequence composition to temporal regulatory dynamics through cascaded second-order transfer functions. This work is a proofof-concept demonstration applied to one representative miRNA and one lncRNA sequence; the framework generates experimentally testable hypotheses and establishes qualitative scaling principles, but quantitative prediction of regulatory dynamics across ncRNA classes will require integration of thermodynamic secondary structure parameters, epitranscriptomic context, and nonlinear feedback effects. Read alongside the companion endocrine framework (2), the two papers together delineate a two-tier frequency hierarchy spanning the full chain from hormonal signal to epigenetic response: the cortisol–HPA axis operates at *τ ≈* 130* min* ultradian period *∼* 90* min*, while the lncRNA-mediated chromatin layer responds on the faster 8–24 minute timescale predicted here. For effective hormonal signal transduction into epigenetic change, the bandwidth of the lncRNA tier must overlap with the input frequency of the hormonal tier—a constraint that the present framework makes quantitatively explicit and experimentally testable. The framework predicts that RNA length and cascade topology encode tunable low-pass filtering properties, with longer ncRNA sequences exhibiting lower cutoff frequencies, greater phase accumulation, and stronger high-frequency attenuation than short miRNAs. The predicted response timescales (8–24 minutes) are broadly consistent with experimentally observed timescales of chromatin remodelling, transcriptional bursting, and RNA-mediated gene regulation— consistency now corroborated by recent experimental studies directly measuring lncRNA-mediated epigenetic dynamics (26) and demonstrating frequency-selective filtering in transcriptional regulation (27, 28). The distinction between G-type (fast, responsive) and H-type (slow, stable) cascade architectures provides a theoretical basis for understanding how the same sequence composition can support distinct regulatory strategies.

## Data Availability

The original contributions presented in the study are included in the article/supplementary material. Further inquiries can be directed to the corresponding author.

## Appendix

**System Definition**

The state vector is **x** = [*x*_1_*,x*_2_]*^T^* = [*S,C*]*^T^*, i.e. *x*_1_ and *x*_2_ are the state-space labels for *S* and *C* respectively

(Equations 1, 2), *u* is the input signal (corresponding to *R_a_*), and *s* below is the Laplace-transform variable, as defined in the Methods. The matrices *A* and *B* for the system are:

(10)
$$A=[\begin{array}{cc} 0 & -k_{3}\\ k_{4} & -k_{2} \end{array}],\;B=[\begin{array}{c} 1\\ 0 \end{array}]$$

**Transfer Functions**

Characteristic equation polynomial:

(11)
$$\text{det }(sI-A)=s(s+k_{2})+k_{3}k_{4}$$

Individual transfer functions to x1, to flux k3x1, to x2, and to flux k2x2:

(12)
$$\frac{X_{1}(s)}{U(s)}=\frac{s+k_{2}}{s(s+k_{2})+k_{3}k_{4}}$$

(13)
$$\frac{k_{3}(s+k_{2})}{s(s+k_{2})+k_{3}k_{4}}$$

(14)
$$\frac{X_{2}(s)}{U(s)}=\frac{k_{4}}{s(s+k_{2})+k_{3}k_{4}}$$

(15)
$$\frac{k_{2}k_{4}}{s(s+k_{2})+k_{3}k_{4}}$$

**Networks**

**Case 1:** *k*_3_*x*_1_ flux network of *N* stages:

(16)
$$G_{i}(s)=\frac{k_{3i}(s+k_{2i})}{s(s+k_{2i})+k_{3i}k_{4i}}$$

(17)
$$\frac{Y_{N}(s)}{U_{1}(s)}=\frac{\prod_{j=1}^{N}k_{3,n_{j}}(s+k_{2,n_{j}})}{\prod_{j=1}^{N}[s(s+k_{2,n_{j}})+k_{3,n_{j}}k_{4,n_{j}}]}$$

**Case 2:** *k*_2_*x*_2_ flux network of *N* stages:

(18)
$$G_{i}(s)=\frac{k_{2i}k_{4i}}{s(s+k_{2i})+k_{3i}k_{4i}}$$

(19)
$$\frac{Y_{N}(s)}{U_{1}(s)}=\frac{\prod_{j=1}^{N}k_{2,n_{j}}k_{4,n_{j}}}{\prod_{j=1}^{N}[s(s+k_{2,n_{j}})+k_{3,n_{j}}k_{4,n_{j}}]}$$
